# Physical Activity versus Psychological Stress: Effects on Salivary Cortisol and Working Memory Performance

**DOI:** 10.3390/medicina55050119

**Published:** 2019-04-30

**Authors:** Pamela Ponce, Alberto del Arco, Paul Loprinzi

**Affiliations:** 1 Exercise and Memory Laboratory, Department of Health, Exercise Science and Recreation Management, The University of Mississippi, University, MS 38677, USA; splam07@gmail.com; 2 Neurophysiology and Behavior Laboratory, Department of Health, Exercise Science and Recreation Management, The University of Mississippi, University, MS 38677, USA; adelarco@olemiss.edu

**Keywords:** exercise intensity, social stress, HPA axis, executive functions, glucocorticoids, humans

## Abstract

*Background and Objective*: The present study was designed to investigate whether acute physical activity and psychological stress produce different effects on cortisol release and working memory performance. *Materials and Methods*: Male subjects (*N* = 12; 18–35 years) were recruited and scheduled to come four times to our lab (within-subject design). For each counterbalanced visit, they performed one of the following four protocols: control, moderate physical activity (MOD), vigorous physical activity (VIG), and acute stress. Heart rate was monitored during every protocol. MOD and VIG were performed for 15 min and were defined as 40–50% and 70–80%, respectively, of their maximum heart rate. Acute stress was imposed via the Trier Social Stress Test (TSST). Salivary samples were collected before and after every protocol to assess cortisol concentrations. Working memory (WM) performance was evaluated through the 2N-Back task right after ending the protocol (early WM) and after a delay of 35 min (late WM). *Results*: VIG and stress, but not MOD, increased salivary cortisol concentrations. However, the increases of cortisol produced by VIG and stress were not significantly different. Also, there were no significant differences in working memory performance (late and early) in any of the experimental protocols tested. *Conclusions*: These results show that exercise (VIG) and stress produce similar effects on cortisol release and do not support the hypothesis that working memory capacity is influenced by elevated cortisol levels, either from varying exercise intensities or psychological stress.

## 1. Introduction

Physical activity and psychological stress modulate memory function. However, studies in humans and animals show that exercise and stress may produce different effects on memory [1]. Acute exercise, particularly moderate-intensity exercise, may enhance working memory capacity (Weng, Pierce, Darling, Voss [2]). In contrast, acute stress seems to impair working memory performance [1,3]. Yet, how exercise and stress can produce opposite effects on working memory function is not fully understood.

The positive effects of exercise are multifold, and in part, may be attributed to increased arousal and executive control [4,5,6,7]. In particular, the effects of exercise on working memory capacity depend on the intensity of exercise. Moderate-intensity exercise may be most advantageous to improve working memory [8,9]. However, this may vary depending on the outcome metric for working memory (i.e., reaction time or accuracy) [10]. The potential negative effects of acute stress on memory have been related, in part, to increased cortisol concentrations as well as catecholamines, which can affect neuronal excitability and alter the brain mechanisms involved in working memory (i.e., prefrontal cortex function) [11,12]. Importantly, acute physical activity also increases cortisol [13], which challenges the potential differential effects of exercise and stress on working memory, as well as the role that cortisol may play in these relationships. This conundrum has recently been referred to as the Exercise-Glucocorticoid Paradox [14].

Given that both exercise and stress stimulate the release of cortisol, different factors may contribute to their differential impact in working memory. First, physical activity-induced increases of cortisol depend on intensity. In fact, vigorous (60–80% maximum capacity), but not moderate (40–50% maximum capacity), exercise increases cortisol levels [15]. Therefore, it is possible that moderate exercise improves working memory because it does not substantively increase cortisol concentrations. Second, cortisol takes several minutes to reach its peak increase after acute bouts of exercise or stress [16]. The effects of exercise, and stress, on working memory performance may depend on the time course of cortisol increases [12,17]. Therefore, it is possible that exercise and stress produce a different time course of cortisol release which in turn could produce different time-dependent effects on working memory performance. This can make a difference when the effects of acute exercise or stress are tested immediately after or within a delay.

The present study was designed to compare acute physical activity and psychological stress and determine whether these experimental manipulations produce different effects on cortisol increases and working memory performance. To our knowledge, no such investigation exists. We investigated the effects of different intensities of exercise, moderate (MOD) and vigorous (VIG), and stress using a within-subject experimental design in which every subject was exposed to 15-min bouts of exercise, stress and control conditions. Salivary cortisol was collected at different time points before and after the experimental manipulations. Working memory performance (2N-Back) was evaluated at two points, immediately and 35 min after the end of these manipulations. Heart rate and psychological effects (positive and negative affect schedule (PANAS) questionnaire) of exercise and stress were also evaluated.

## 2. Methods and Protocols

### 2.1. Study Design and Participants

This study was approved by the authors’ institutional review board (No. 17-086) and all participants provided written consent prior to participation. Data collection occurred between the Spring of 2018 and mid-Summer of 2018. The present experiment was a within-subject, counterbalanced study design. Participants completed four separate visits, each occurring approximately 48–72 h apart. Further details on these visits are described below. In brief, 12 male participants (college students), which is similar in sample size to other related experiments [18,19], were recruited via a non-probability sampling approach (classroom announcements and word-of-mouth). This is based on an anticipated sample size of 10 needed to achieve adequate statistical power (1-β error probability) of 0.80, based on inputs of 0.05 (α error probability), four visits with two memory assessments per visit, and an effect size of 0.10 (η2p). Male only subjects were evaluated to remove any potential confounding effects of the menstrual cycle on working memory capacity and cortisol levels [20,21,22]. Further, we focused on males only because males tend to have a greater increase in cortisol from stress-induction protocols [23,24].

### 2.2. Experimental Conditions

**Figure 1:**
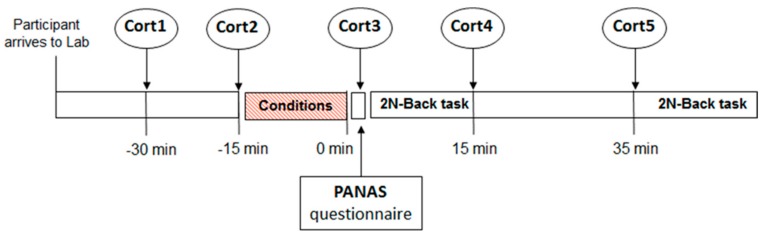
Timeline of the experimental protocol used in the study. Two baseline cortisol samples were collected (Cort1 and 2). Then, the experimental condition started-control, moderate exercise (MOD), vigorous exercise (VIG), and psychological stress, in counterbalanced order, and ended after 15 min. Following this, a third cortisol sample was obtained (Cort3) and the positive and negative affect schedule (PANAS) questionnaire was filled out by subjects. Participants completed the 2N-Back working memory task immediately after (early working memory (WM)) and 35 min after (late WM) the end of the experimental condition. Also, a fourth and fifth cortisol samples (Cort4 and 5) were collected at 0 min and 35 min, respectively.

Figure 1 displays the study protocol for each of the experimental conditions. Two baseline cortisol samples were obtained (15-min apart). Following the second resting baseline cortisol sample, the experimental manipulation occurred-control, moderate exercise (MOD), vigorous exercise (VIG), and psychological stress, in a counterbalanced order. Following the experimental manipulation, a third cortisol sample was obtained and the PANAS survey (see below) was filled out by participants. Immediately thereafter, they completed the 2N-Back working memory task (early working memory (WM)), with a fourth and fifth cortisol sample, respectively, occurring 15-min and 35-min thereafter. Participants completed the 2N-Back task again (late WM) after the fifth cortisol sample.

All experimental manipulations lasted 15-min, with heart rate (Polar, F1) assessed throughout. The control visit involved playing an on-line game (e.g., Sudoku), which has been employed in other related experiments [25]. The moderate-intensity treadmill exercise involved exercising (walking) at approximately 60% of estimated (220-age) heart rate max. The vigorous-intensity treadmill exercise involved a progressive bout of near-maximal jogging exercise, identical to our previous work [26]. Regarding the psychological stress induction, modeled after the Trier Social Stress Test [27], participants completed two stress-inducing tasks. Participants were first told to sit quietly and prepare for a speech (i.e., being interviewed for their dream job) that they would be delivering to the research team. They were told that this speech would be recorded and shown to University faculty for review of public speaking strengths and weaknesses (this never actually happened). Additionally, they were told that they could use their notes during their presentation, but then right before the presentation, the notes were taken away from the participant. After 10-min of preparation, they delivered the video-recorded speech during 10 min. Two researchers were always present during this time which consisted of a 5-min speech followed by a 5-min arithmetic task that involved sequentially subtracting 17 from the number 2023.

### 2.3. Survey

To assess mood status, immediately after each experimental manipulation (control, MOD, VIG, and psychological stress), participants completed the Positive and Negative Affect Schedule (PANAS) [28]. For this mood survey, participants rated 20 items (e.g., excited, upset, irritable, and attentive) on a Likert scale (1, very slightly or not at all; to 5, extremely), with half of the items constituting a “positive” mood state, with the other half being a “negative” mood state. For each subscale (positive/negative mood), items were summed, with higher scores indicative of a higher affective state (i.e., more positive, more negative affective state).

### 2.4. Working Memory

Working memory capacity was evaluated using a 2N-Back computer task, utilizing CogState software (https://www.cogstate.com/). This task, lasting approximately 3-min, involved viewing poker cards, displayed one at a time, tasked with trying to remember two cards back. The proportion of correct responses (accuracy) was evaluated from the Arcsine transformation, which is the square root of the proportion of correct responses (higher score indicates better performance). Reaction time was expressed as the mean of the log_10_ transformed reaction times for the correct responses and expressed as log_10_ milliseconds.

### 2.5. Cortisol

Salivary cortisol was collected using the Sarstedt Cortisol Salivette Device (Sarstedt, Numbrecht, Germany) in a Salivette tube. Participants chewed on a round sponge for 60-s and then deposited the sponge and saliva directly into the tube. Participants did not brush their teeth, floss, or consume food or water for at least 60-min prior to the assessment. Samples were immediately refrigerated (−20 degrees Celsius), and then within a few hours, were centrifuged (1000× *g*/2 min), followed be immediate storage (−80 degrees Celsius). Cortisol concentrations in the saliva were quantified using the R and D Cortisol Parameter Assay (R and D Biosciences, Minneapolis, MN, USA). Per manufacturer’s recommendations, the samples were diluted 5-fold and run in technical duplicates.

### 2.6. Data and Statistical Analysis

Salivary cortisol concentrations are reported as absolute values in ng/mL. Participants were divided into two groups: cortisol increase group (CI), if they showed increases of cortisol ≥2.5 nmol/L (0.9 ng/mL) [17] in at least two of the three samples collected after the end of VIG or stress; and no-cortisol increase group (NCI). Statistical analyses were computed in SPSS v. 24 (IBM Corp., Armonk, NY, USA). Data approximated normality, and thus, parametric inferential statistics were employed. Repeated measures ANOVA (analysis of variance) were employed, evaluating differences in the outcome measures (e.g., heart rate, mood, cortisol, and working memory) across the experimental conditions. Independent and Paired *t* tests were also used to further compared cortisol and working memory accuracy among groups. Partial eta-squared (η2p) effect size estimates were calculated. Statistical significance was established as 0.05.

## 3. Results

### 3.1. Participants

Table 1 displays the characteristics of the sample. Participants, on average, were 25.0 years, 100% were male, with variability across race-ethnicity (58.3%, 16.7%, and 25.0%, respectively, were white, black, and other). The sample, on average, was of a normal body mass index (*M*_BMI_ = 25.7 kg/m^2^) and was fairly active (235 min/week of moderate to vigorous physical activity (MVPA)).

### 3.2. Physiological and Behavioral Measures

Table 2 displays the physiological (heart rate) and psychological (affect) responses, and working memory performance for each of the experimental conditions (Control, MOD, VIG, and psychological stress).

Heart Rate: Resting heart rate was similar across the four experimental conditions (F(3,33) = 0.28, *p* = 0.84, η2p = 0.02). The moderate-intensity exercise protocol significantly increased heart rate from 69.2 bpm to 113.7 bpm (F(1,11) = 60.8, *p* < 0.001, η2p = 0.85). The vigorous-intensity exercise protocol also significantly increased heart rate, but to a greater extent, from 70.8 bpm to 181.9 bpm (F(1,10) = 573.8, *p* < 0.001, η2p = 0.98). Similarly, the psychological stress-induction also significantly increased heart rate, from 73.0 bpm to 89.5 bpm (F(1,11) = 11.91, *p* = 0.005, η2p = 0.52).

Psychological Responses (PANAS): The affective response was different depending on the experimental condition. The two exercise conditions elicited the greatest positive affect response, whereas the psychological stress-induction had the lowest positive affective state (F(3,33) = 4.41, *p* = 0.01, η2p = 0.29). Similarly, the two exercise conditions had the lowest negative affective state, whereas the psychological stress-induction condition resulted in the highest negative affective state (F(3,33) = 11.63, *p* < 0.001, η2p = 0.51).

Working Memory Performance: There were no significant differences in working memory accuracy across the experimental conditions, as noted by a non-significant main effect for condition (F(3,42) = 0.65, *p* = 0.588, η2p = 0.04) and time (F(1,42) = 0.50, *p* = 0.481, η2p = 0.01). None of the four conditions changed working memory reaction time, as noted by a non-significant condition by time interaction (F(3,33) = 0.16, *p* = 0.92, η2p = 0.01).

### 3.3. Salivary Cortisol 

**Figure 2:**
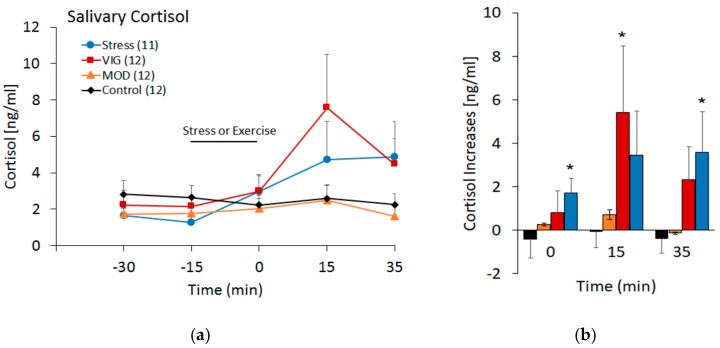
Effects of the four experimental conditions (solid line)—control, moderate exercise (MOD), vigorous exercise (VIG), and psychological stress—on cortisol concentrations. (**a**) Temporal profile of cortisol increases. Data are the mean ± SE of the absolute values (ng/mL) of salivary cortisol. (**b**) Increases of cortisol at 0 min, 15 min, and 35 min after the end of the experimental conditions. Data are the mean ± SE of the absolute values (ng/mL) at these time points after the subtraction of the second baseline sample (−15 min). Number of subjects in parenthesis. * *p* < 0.05 compared to control after paired *t* test.

Figure 2 shows the effects of exercise, MOD and VIG, and psychological stress on salivary cortisol. As shown in Figure 2A, both VIG and stress increased the salivary concentrations of cortisol (F(4,43) = 5.10, *p* = 0.01, η2p = 0.10) as a main effect for time. This same analysis did not detect statistical differences among conditions (F(3,43) = 1.35, *p* = 0.27, η2p = 0.08). Figure 2B shows the results of a further analysis in which the increases of cortisol (after subtracting the baseline) for all the experimental conditions are compared at different time points (0 min, 15 min, and 35 min) after the end of the experimental manipulation. Cortisol increases produced by stress were significantly different from control at 0 min (t(43) = 2.06, *p* = 0.045) and 35 min (t(43) = 2.33, *p* = 0.024, paired *t* test), while the increases produced by VIG were different from control at 15 min (t(43) = 2.13, *p* = 0.038, paired *t* test). Cortisol increases after VIG and stress were not significantly different at any time point. Similarly, MOD did not produce significant increases of cortisol at any time point.

**Figure 3:**
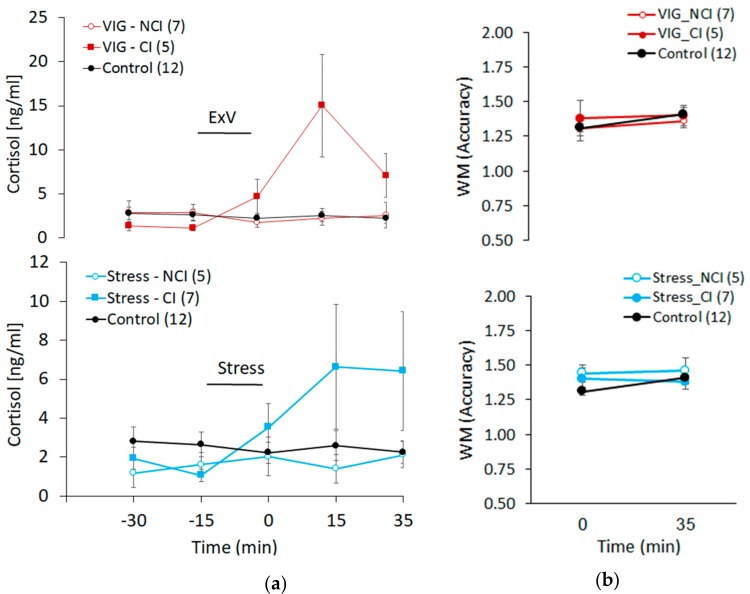
Effects of VIG and stress on cortisol (**a**) and working memory performance (**b**) after grouping subjects according to whether these experimental manipulations increase (CI) or not (NCI) cortisol concentrations. (**a**) Temporal profile of cortisol increases. Data are the mean ± SE of the absolute values (ng/mL) of salivary cortisol. (**b**) Early WM (0 min) and late WM (35 min) after VIG and stress. Data are the mean ± SE of the absolute values of accuracy. Number of subjects in parenthesis. * *p* < 0.05 compared to control after independent *t* test.

Cortisol and Working Memory: To further evaluate the relationship between cortisol increases and working memory performance, participants were divided into two groups depending on whether they showed cortisol increases (CI group), or not (NCI group), after VIG and stress. Specifically, and according to previous studies [17,29], participants showing increases of cortisol ≥ 2.5 nmol/L (0.9 ng/mL) in at least two of the three samples collected after VIG or stress were classified as CI subjects. As shown in Figure 3A, the average increases of cortisol after VIG (t(21) = 3.59, *p* = 0.002, independent *t* test) and stress (t(21) = 2.37, *p* = 0.027, independent *t* test) in the CI group were significantly different from control. However, as shown in Figure 3B, working memory performance in the CI group after VIG (early WM: t(14) = 0.91, *p* = 0.376; late WM: t(15) = 0.62, *p* = 0.546, independent *t* test) or stress (early WM: t(16) = 1.39, *p* = 0.181; late WM: t(16) = 0.38, *p* = 0.706, independent *t* test) was not different from control, which suggest that cortisol increases did not modulate working memory performance at any of the time points evaluated.

## 4. Discussion

The present study examined whether acute physical activity and psychological stress produce different effects on salivary cortisol and working memory performance. The results show that, first, VIG, but not MOD, and stress increase salivary concentrations of cortisol. The increases of cortisol produced by VIG and stress were not statistically different. Second, working memory performance (2N-back) was not changed by exercise or stress, measured either immediately or within a delay after the end of these experimental manipulations. Moreover, grouping subjects according to their cortisol increases (CI versus NCI) showed that working memory performance is not associated with cortisol increases after exercise or stress. These results do not provide evidence that cortisol increases influence working memory function after these experimental manipulations. Further, this study also suggests that cortisol increases do not explain potential different effects of exercise (VIG) and stress on working memory-related executive functions.

As shown, both acute exercise and stress increased salivary concentrations of cortisol. In particular, 15-min exercise produced an intensity-related effect on cortisol. These results agree with previous studies that show increases of cortisol as a function of both exercise intensity and duration [13]. We also show that acute psychological stress (Trier social stress test; TSST) increases cortisol concentrations which also agrees with previous studies using the same or different stress protocols [3,17]. Importantly, our study compares the increases of cortisol produced by acute exercise and stress in the same sample and shows that the temporal profile and the average cortisol increases were not statistically different in both groups (VIG and stress). These results suggest that exercise (VIG) and stress do not produce different effects on cortisol increases. Also, these results are relevant to compare the effects of exercise and stress on working memory function.

Previous studies in humans and animals have shown that cortisol increases are associated with working memory impairments after acute stress [3,11]. In this study, we investigated whether cortisol increases produced by physical activity and stress were associated with different changes in working memory performance. To evaluate a potential time effect of cortisol increases, working memory was evaluated at two time points, immediately (early WM) and 35 min (late WM), after the end of these experimental manipulations. As shown in Table 2, working memory performance (i.e., reaction time and accuracy) was not significantly changed by exercise (MOD and VIG) or stress either immediately or after a 35 min delay. The same results were observed after grouping participants according to their cortisol increases, supporting the fact that cortisol is not sufficient to impair or change working memory performance regardless whether these increases are produced by exercise or stress.

Our null findings regarding the relationship between changes in cortisol and working memory capacity align with recent empirical work among humans [30]. These null findings have occurred in samples of men [31,32,33] and women [34,35]. There are various factors that may influence whether or not cortisol has an impact on working memory function. For instance, the impairment effects of cortisol on working memory may depend on concurrent elevations of norepinephrine in the amygdala [24]. Also, the time of day, the degree of stress induction or the difficulty of the task may influence the effects of cortisol on working memory capacity [29,36]. Recent work in humans has also shown that working memory performance may vary or be influenced by a multitude of other demographic, behavioral, and psychological parameters [37]. Further, the complexity of the cortisol-working memory relationship is illustrated by other situations where elevated cortisol may actually enhance working memory [38].

We anticipated that MOD and VIG would produce different effects on working memory function. However, as shown, none of our evaluated exercise intensities changed working memory performance despite producing different sympathoadrenal activation (heart rate increase) and salivary cortisol increases (only VIG increased cortisol). In line with these results, some studies show that varying exercise intensities are not associated with working memory changes [39,40,41]. In contrast, other studies showed that moderate-intensity exercise [2,42,43] and vigorous-intensity [44,45] exercise may enhance working memory. Interestingly, Li et al. [7] demonstrated that acute exercise changes working-memory at the neural level (increased brain activity), but not at the behavioral level. Therefore, we cannot rule out that MOD and VIG change the neurophysiological mechanisms involved in working memory function without affecting the performance on the 2N-Back task. Additionally, other research demonstrates that individual differences may account for potential effects of exercise on working memory function [46,47].

Strengths of this study include the study novelty, including evaluating the effects of both exercise intensity and psychological stress on working memory function in the same sample. Further, we measured physiological (cortisol and heart rate) and psychological (affect) responses to each of the experimental manipulations. A limitation of this study includes the relatively small sample among a homogenous sample (male college students), which precludes the ability to evaluate potential moderators (e.g., individual differences).

## 5. Conclusions

In summary, this study shows that both exercise (vigorous) and stress do not change working memory performance despite producing similar increases in salivary cortisol. These results do not support a role of cortisol increases modulating working memory function after acute exercise or stress. Moreover, these results do not support a potential role for cortisol in the different effects of exercise (VIG) and stress on working memory observed in previous studies. Clearly, future work on this topic is needed. If such work demonstrates a differential effect of acute exercise and stress on working memory capacity, then effective coping strategies will need to be identified and implemented to attenuate such stress-induced working memory impairment effects [48].

## Figures and Tables

**Table 1:** Demographic and behavioral characteristics of the sample (*N* = 12).

Age, Mean Years	25.0 (4.2)
Gender, % male	100.0
Race-Ethnicity, %	
Non-Hispanic White	58.3
Non-Hispanic Black	16.7
Other	25.0
Education, %	
Undergraduate Student	41.7
Graduate Student	58.3
Body Mass Index, mean kg/m^2^	25.7 (3.3)
Waist Circumference, mean cm	90.7 (11.2)
Physical Activity, mean MVPA/week	235.0 (100.3)

MVPA: Moderate to vigorous physical activity, Standard deviation in parenthesis.

**Table 2:** Physiological (heart rate), psychological (affect), and cognitive (working memory) responses to the experimental conditions (*N* = 12). Dashed line (-) indicates measurement was not taken. Standard Deviation in parenthesis.

Variable	Control	Moderate Exercise (MOD)	Vigorous Exercise (VIG)	Psychological Stress
**Heart Rate, mean beats per minute**				
Rest	71.6 (11.8)	69.2 (14.6)	70.8 (13.3)	73.0 (11.3)
Mid-Point	-	110.8 (16.5)	161.4 (19.1)	89.5 (17.5)
End	-	113.7 (16.2)	181.9 (11.0)	85.6 (13.6)
5-Min Post	-	76.2 (15.9)	105.2 (15.0)	-
**Affect, Post Stimulus**				
Positive PANAS, mean	22.5 (8.9)	25.2 (8.6)	30.2 (8.0)	21.6 (5.2)
Negative PANAS, mean	12.7 (3.0)	12.2 (2.2)	12.6 (3.0)	22.3 (9.4)
**Cognitive, mean RT (2 N-Back) a**				
Early WM (0 min)	2.90 (0.11)	2.85 (0.08)	2.94 (0.15)	2.88 (0.11)
Late WM (35 min)	2.88 (0.09)	2.82 (0.08)	2.90 (0.12)	2.84 (0.07)
**Cognitive, mean accuracy (2 N-Back) b**				
Early WM (0 min)	1.31 (0.11)	1.39 (0.12)	1.30 (0.22)	1.35 (0.27)
Late WM (35 min)	1.40 (0.17)	1.33 (0.15)	1.35 (0.12)	1.34 (0.27)

PANAS: Positive and Negative Affective Schedule; RT: Reaction Time from 2 N-Back working memory task; a Reaction time expressed as the mean of the log10 transformed reaction times for the correct responses and expressed as log10 milliseconds; b Arcsine transformation of the square root of the proportion of correct responses (higher score indicates better performance) Standard Deviation in parenthesis.
